# The Distribution and Phenotypes of Blood Groups in Hematologic Malignancies

**DOI:** 10.5152/eurasianjmed.2023.21124

**Published:** 2023-02-01

**Authors:** Mete Erdemir, Fuat Erdem, Gülden Sincan

**Affiliations:** 1 Department of Internal Medicine, University of Health Sciences, Erzurum Regional Training and Research Hospital, Erzurum, Turkey; 2 Department of Hematology, Atatürk University, School of Medicine, Erzurum, Turkey

**Keywords:** ABO blood group, duffy blood group, hematologic malignancy, kell blood group

## Abstract

**Objective:**

Blood groups are associated with duodenal ulcer, diabetes mellitus, and urinary tract infection. In some studies, a relationship was detected between hematologic and solid organ malignancies and blood groups. In this study, we investigated the frequency and phenotypes of blood groups (ABO, Kell, Duffy, Rh) in patients with hematologic malignancies.

**Materials and Methods:**

One hundred sixty-one patients with hematologic malignancy (multiple myeloma, chronic lymphocytic leukemia, and chronic myelocytic leukemia) and 41 healthy people were evaluated prospectively. We determined phenotypes and distribution of ABO, Rh, Kell, and Duffy blood groups in all cases. Chi-square test and 1-way variance analysis were used for statistical analysis. *P* < .05 value was considered statistically significant.

**Results:**

In patients with multiple myeloma, the A blood group was statistically significantly more frequent than in the control group (*P* = .021). Rh negativity was found more frequent in patients with hematologic malignancy than the control group (*P* = .009). Kpa and Kpb antigen positivity were found statistically significantly less frequent in patients with hematologic malignancy (*P* = .013, *P* = .007; respectively). Fy (a−b−) and K−k+ phenotypes were higher in patients with hematologic cancer than in the control group (*P* = .045).

**Conclusion:**

We determined a significant relationship between hematologic malignancies and blood group systems. In our study, due to the low number of cases and few hematological malignancy types, extensive studies with more cases and more hematologic cancer types are needed.

**Main Points**

- There is a relationship between blood groups and hematological malignancies.
- Rh negativity is more frequent in patients with hematologic malignancy.
- A blood group is more frequent in patients with multiple myeloma.
- Kpa and Kpb antigen positivity are less frequent in patients with hematologic malignancy.

## Introduction

Blood group antigens are surface markers on erythrocytes.^1^ In addition, these antigens can be found in some tissues and body fluids.^2^ Thirty-six blood group systems and 360 blood group antigens have been identified until today (1). ABO and Rhesus (Rh) blood group systems are the most important blood groups in the clinic.^3^ The distribution of blood group antigens varies according to race and gender. It was reported that the ABO blood group was associated with many diseases such as cognitive impairment, preeclampsia, bleeding, neoplastic diseases, duodenal ulcer, diabetes mellitus, and long life.^2-4^ The Rh blood group system consists of 55 independent antigens and is important in transfusion medicine. Rh positivity is associated with chronic hepatitis, hemolytic disease of the newborn, and hemolytic anemia.^5^

Kell blood group system consists of 36 antigens and K/k (KEL1/KEL2), Kpa/Kpb (KEL3/KEL4), and Jsa/Jsb (KEL6/KEL7) are the most important.^6,7^ Kell antigens are expressed in testicles, brain, and muscle tissue except erythrocytes.^8^ XK protein encoded by the XK gene also plays a role in the expression of Kell antigens. Kell and Duffy blood group systems are associated with hemolytic disease and hemolytic transfusion reaction of the newborn.^9-11^

Duffy blood group system consists of 5 antigens called Fya, Fyb, Fy3, Fy5, and Fy6. This system includes 5 phenotypes as Fy (a+b−), Fy (a−b+), Fy (a+b+), Fy (a−b−), and Fy (a−b +) + (wK).^12^ Duffy blood group antigens act as receptors for cytokines and chemokines. For this reason, they are called DARC (Duffy antigen receptor for chemokine).^13^ Duffy antigen receptor for chemokine has a very important effect on angiogenesis and metastasis in cancer development. Duffy antigen receptor for chemokine expression in endothelial cells leads to aging of these cells and weakening of angiogenesis.^14^ A relationship between DARC and breast cancer, prostate cancer, and non-small cell lung cancer was reported in some studies.^12^

The frequency and distribution of blood group systems may differ in the patients with hematologic malignancy (HM). Therefore, in our study, we aimed to investigate the frequency of blood group subtypes in patients with HM.

## Materials and Methods

### Subjects and Procedures

In this study, 161 patients without a history of bone marrow transplantation and diagnosed with chronic lymphocytic leukemia (CLL), chronic myelocytic leukemia (CML), and multiple myeloma (MM) and 41 healthy people were included in this study. Patients were selected from patients who applied to the department of hematology of Atatürk University Medical Faculty Hospital. The ethics committee of our institution approved the study (B.30.2.ATA.0.01.00/30). An informed consent form was received from the participants. In all cases, anti-K, anti-k, anti-Kpa, anti-Kpb, anti-Fya, anti-Fyb, anti-C, anti-c, anti-E, anti-e, anti-D, anti-A, anti-B antibodies that provided by the Scientific Research Projects Support Office were studied. Anti-k, anti-Kpa, anti-Kpb, anti-Fya, and anti-Fyb were studied manually and anti-C, anti-c, anti-E, anti-e, anti-D, anti-K, anti-A, anti-B, and anti-D were otomatically studied by Galileo Immucor Gamma (Mikroplak, Germany) machine in Blood Center Laboratory of Atatürk University Medical Faculty Hospital.

The tubular method was used as the manual method and 2 mL of venous blood taken from the patients was put into the tube with ethylenediaminetetraacetic acid (EDTA). A drop of blood was dropped into a glass tube via Pasteur pipette from the tube with EDTA. First, the blood was washed 3 times with 0.9% NaCl. Later, it was diluted with 0.9% NaCl until the dark red color was obtained. A drop of diluted blood was dropped into 3 separate glass tubes via Pasteur pipette and 2 drops of Kpa, Kpb, k, Fya, and Fyb antibodies were dropped on each glass tube. Samples with Kpa, Kpb, and k antibodies were kept for 20 minutes, samples with Fya, and Fyb antibodies were kept for 30 minutes in a water bath at 37°C. Samples removed from the water bath were washed 3 times with 0.9% NaCl and 2 drops of NOVACLONE^TM^Anti-IgG, C3d was added to each tube. After that, the samples were centrifuged at 4000 rpm for 1 minute, and agglutination status was evaluated macroscopially or microscopially. The detection of agglutinate macroscopically or presence of 6-8 erythrocyte agglutination in the microscopic examination was evaluated as antigen positive.

### Statistical Analysis

Statistical Package for the Social Sciences software 20.0 (IBM SPSS Statistics for Windows, Version 20.0. Armonk, NY: IBM Corp) package program was used for the analysis of data. Frequency analysis was used to evaluate the demographic characteristics of the cases. Post hoc Duncan analysis was used for multiple comparisons and the relationship between categorical variables was determined with the chi-square independence test. *P* < .05 value was considered statistically significant.

## Results

Fifty-two (32.2%) of the patients with HM were MM, 34 (21.1%) were CML, and 75 (46.7%) were CLL. The mean age was 61.5 ± 12.3 years in the HM group and it was 59.2 ± 14 years in the control group. The gender distribution of patients with HM and the control group is shown in Table 1. There was no statistically significant difference between HM and control groups in terms of age and gender distribution. Distribution and phenotypes of ABO, Rh, Kell, and Duffy blood groups in MM, CML, CLL, HM, and control groups are given in Table 2.

The control group and HM (CML, CLL, and MM) group were compared in terms of ABO, Rh, Kell, Duffy, C, c, E, e blood groups and Duffy, kell, Rh blood group phenotypes. Significant differences between the control group and HM, MM, CLL, and CML for these parameters are shown in Tables 3-6 respectively. In the comparison of the control group and HM, MM, CML, and CLL groups, there was no significant difference except for the parameters that are shown in the tables.

## Discussion

It has been reported that there is a relationship between the ABO blood group and breast, stomach, pancreas, ovarian, nasopharynx, colorectal, and esophagus cancers, but the effects of blood groups on cancer development are not fully known. The blood group antigens are expressed from the surface of malign cells and these antigens are different from blood group antigens expressed in normal erythrocytes. Modified blood group antigens expressed from the surface of cancer cells may affect cancer development and spread by changing cell mobility and apoptosis sensitivity and escape from the immune system.^15,16^ It was reported that the cancer incidence was higher in people with A blood group than in people with non-A blood group in a meta-analysis conducted by Zhang et al^15^ between 1953 and 2013. In the same study, the overall cancer incidence was lower in the person with O blood group than in the person with other blood groups.^15^ In another study, A blood group was determined as 43.9%, B blood group as 17.3%, AB blood group as 8%, and O blood group as 30.8% in 1055 patients with acute and chronic leukemia.^17^ In accordance with the literature, we detected the most common blood group was A blood group in patients with HM. However, in our study, the O blood group was found to be the second highest group in the HM group and it was not compatible with the literature.

Blood group distribution in CML patients was as follows: O blood group was 43%, A blood group was 36.3%, B blood group was 7.2%, and AB blood group was 2.9%.^18^ In a study, 2579 patients were diagnosed with hematological malignancy in Iran; 37% of the patients with CML were of O blood group, 34% were of A blood group, 23% were of B blood group, 6% were of AB blood group.^19^ B blood group frequency was high and the frequency of O blood group was low in patients with CML in another study.^20^ Kar et al^17^ identified that O blood group was 31.2%, A blood group was 49.6%, B blood group was 14.9%, and AB blood group was 4.3% in patients with CML in Turkey. In our study, the O blood group was the most common blood group in CML patients. This result is compatible with studies in Iran.

Kar et al^17^ reported that the A blood group was the most common blood group in CLL patients in Turkey. But Novaretti et al^18^ detected the most common blood group was the O blood group in CLL patients. In addition, 34% A blood group, 18% B blood group, 43% O blood group, and 5% AB blood group were detected in CLL patients in Iran.^19^ In our study, the most common blood type in CLL cases was A blood group. This result is consistent with the study conducted in Turkey.

Distribution of A, B, AB, and O blood groups in MM patients were 36%, 12%, 8%, and 44%, respectively, in a study that was made in the European Institute of Oncology.^21^ In a study conducted in Iran, O blood group was the most common blood group in multiple myeloma patients.^19^ In our study, the A blood group was the most common blood type in patients with MM. This result was in accordance with the literature.

The relationship between Rh blood group and cancer development has been investigated. About 3944 breast cancer patients were examined in Turkey and 88.2% of the patients were Rh (+) and 11.8% were Rh (−).^22^ The frequency of invasive lobular breast cancer was higher in Rh (+) patients than in patients with Rh (−) in another study examining 209 breast cancer cases.^23^ In a study conducted in Tehran University in Iran, 96% of MM patients were Rh positive and 4% were Rh negative.^19^ In our study, Rh negativity was higher in patients with HM than the control group. Comparison with the literature could not be made as there are no studies examining the relationship between hematologic cancers and Rh blood group.

The E antigen was most often detected in patients with CML but was not statistically significant and e antigen was statistically significantly higher in patients with CLL compared to the control group. In patients with MM, the ccdee phenotype was found higher than the control group in our study. But, there were no studies investigating the relationship between Rh subgroups and solid, hematologic cancers. Therefore, our study results could not be compared with the literature.

Duffy blood group antigens act as receptors for cytokines and chemokines. These chemokines play a role in breast, prostate, non-small cell lung cancers, and MM pathogenesis.^12,13^ In our study, although Fya and Fyb antigens were seen less frequently in patients with HM compared to the control group, there was no statistically significant difference between HM and control groups. About 5022 female patients with benign or malign breast diseases were examined in Shanghai Cancer Hospital. Fy (a−b−) phenotype was found most frequently in patients with breast cancer.^12^ In our study, only Fy (a−b−) phenotype was found statistically significantly high in patients in the HM group. Güler et al^13^ detected that 77% of MM patients were Fy (a+b+) phenotype in Turkey. In our study, the Fy (a+b+) phenotype was the most common phenotype in patients with HM and MM, but this difference was not statistically significant.

Our study had some limitations. Our study group consisted of relatively few cases. The distribution of blood groups may differ between societies. Our study is a single-center study. Therefore, the results may not be representative, and multicenter prospective studies are needed.

In conclusion, the presence of blood groups antigens in many tissues except erythrocytes and their association with some diseases are important for the continuation of these researches. Determining the relationship between blood groups and diseases can be helpful for pre-disease protective measures and early diagnosis. Although there are many studies investigating the relationship between solid malignancies and ABO blood groups, there are few studies investigating the relationship between hematologic cancers and blood groups. With this aspect, we think that our study will support the literature.

## Figures and Tables

**Table 1.** Gender Distribution of Patients with HM and Control Groups

Diagnosis	Male, n (%)	Female, n (%)
HM group	102 (63.4)	59 (36.6)
MM	30 (57.7)	22 (42.3)
CML	20 (58.8)	14 (41.2)
CLL	52 (69.3)	23 (30.7)
Control group	21 (51.2)	20 (48.8)

HM, hematologic malignancy; CLL, chronic lymphocytic leukemia; CML, chronic myelocytic leukemia; MM, multiple myeloma.

**Table 3.** Significant Differences Between HM and Control Groups in Terms of ABO, Rh, Kell, Duffy, C, c, E, e Blood Groups and Duffy, Kell, Rh Blood Group Phenotypes

Blood Groups and Phenotypes	HM, n (%)	Control, n (%)	*P*
Rh (+)	124 (77)	39 (95.1)	.009
Rh (−	37 (23)	2 (4.9)	.009
e	157 (97.5)	36 (87.8)	.018
ccdee	30 (18.6)	2 (4.9)	.031
Fy (a−b−)	15 (9.3)	0 (0)	.045
Kpa	24 (14.9)	13 (31.7)	.013
Kpb	117 (72.7)	38 (92.7)	.007
Kp (a+b+)	18 (11.2)	11 (26.8)	.011
Kp (a−b−)	38 (23.6)	1 (2.4)	.002
K−k+	146 (90.7)	41 (100)	.045

HM, hematologic malignancy.

**Table 4.** Significant Differences Between MM and Control Groups in Terms of ABO, Rh, Kell, Duffy, C, c, E, e Blood Groups and Duffy, Kell, Rh Blood Group Phenotypes

Blood Groups and Phenotypes	MM, n (%)	Control, n (%)	*P*
A	29 (55.7)	13 (31.7)	.021
O	12 (23)	18 (43.9)	.033
Rh (+)	38 (73.1)	39 (95.1)	.005
Rh (-)	14 (26.9)	2 (4.9)	.005
ccdee	10 (19.2)	2 (4.9)	.040
Fy (a−b−)	8 (15.4)	0 (0)	.008
Kpb	33 (46.5)	38 (53.5)	.001
Kp (a−b−)	16 (30.8)	1 (2.4)	.000
k	45 (86.5)	41 (100)	.016
K−k+	43 (82.6)	41 (100)	.005
K−k−	7 (13.4)	0 (0)	.016

MM, multiple myeloma.

**Table 5.** Significant Differences Between CLL and Control Groups in Terms of ABO, Rh, Kell, Duffy, C, c, E, e Blood Groups and Duffy, Kell, Rh Blood Group Phenotypes

Blood Groups and Phenotypes	CLL, n (%)	Control, n (%)	*P*
Rh (+)	60 (80)	39 (95.1)	.028
Rh (−)	15 (20)	2 (4.9)	.028
e	75 (100)	36 (87.8)	.002
Fy (a−b−)	7 (9.3)	0 (0)	.050
Kpa	9 (12)	13 (31.7)	.010
Kpb	53 (70.7)	38 (92.7)	.006
Kp (a+b+)	6 (8)	11 (26.8)	.006
Kp (a−b−)	19 (25.3)	1 (2.4)	.002

CLL, chronic lymphocytic leukemia.

**Table 6.** Significant Differences Between CML And Control Groups in Terms of ABO, Rh, Kell, Duffy, C, c, E, e Blood Groups and Duffy, Kell, Rh Blood Group Phenotypes

Blood Groups and phenotypes	CML, n (%)	Control, n (%)	*P*
Rh (+)	26 (76.5)	39 (95.1)	.036
Rh (−)	8 (23.5)	2 (4.9)	.036
Kpa	9 (12)	13 (31.7)	.010
Kpb	53 (70.7)	38 (92.7)	.006
Kp (a+b+)	6 (8)	11 (26.8)	.006
Kp (a−b−)	19 (25.3)	1 (2.4)	.002

CML, chronic myelocytic leukemia.

**Table 2.** Distribution of ABO, Kell, Duffy, Rh Blood Groups and Phenotypes in HM and Control Groups

Blood Groups and Phenotypes	MM, n (%)	CLL, n (%)	CML, n (%)	HM, n (%)	Control, n (%)
A	29 (55.7)	33 (44)	9 (26.5)	71 (44.1)	13 (31.7)
B	8 (15.3)	12 (16)	11 (32.4)	31 (19.3)	7 (17.1)
AB	3 (5.7)	5 (6.7)	3 (8.8)	11 (6.8)	3 (7.3)
O	12 (23.3)	25 (33.3)	11 (32.4)	48 (29.8)	18 (43.9)
Rh (+)	38 (73.1)	60 (80)	26 (76.5)	124 (77)	39 (95.1)
Rh (−)	14 (26.9)	15 (20)	8 (23.5)	37 (23)	2 (4.9)
C	36 (69.2)	49 (65.3)	17 (50)	102 (63.4)	29 (70.7)
c	41 (78.8)	57 (76)	29 (85.3)	127 (78.9)	34 (82.9)
E	17 (32.7)	15 (20)	12 (35.3)	44 (27.3)	12 (29.3)
e	50 (96.2)	75 (100)	32 (94.1)	157 (97.5)	36 (87.8)
CcDee	13 (25)	22 (29.3)	10 (29.4)	45 (28)	16 (39)
CcDEe	12 (23.1)	7 (9.3)	2 (5.9)	21 (13)	4 (9.8)
CDee	9 (17.3)	18 (24)	4 (11.8)	31 (19.3)	6 (14.6)
ccdee	10 (19.2)	13 (17.3)	7 (20.6)	30 (18.6)	2 (4.9)
ccDEe	4 (7.7)	8 (10.7)	6 (17.6)	18 (11.2)	6 (14.6)
Fya	36 (69.2)	52 (69.3)	24 (70.6)	112 (69.6)	31 (75.6)
Fyb	38 (73.1)	49 (65.3)	21 (61.8)	108 (67.1)	32 (78)
Fy (a+b+)	30 (57.7)	33 (44)	11 (32.4)	74 (46)	22 (53.7)
Fy (a−b+)	8 (15.4)	16 (21.3)	10 (29.4)	34 (21.1)	10 (24.4)
Fy (a+b−)	6 (11.5)	19 (25.3)	13 (38.2)	38 (23.6)	9 (22)
Fy (a−b−)	8 (15.4)	7 (9.3)	0 (0)	15 (9.3)	0 (0)
Kpa	9 (40.9)	9 (12)	9 (12)	24 (14.9)	13 (59.1)
Kpb	33 (46.5)	53 (70.7)	53 (70.7)	117 (72.7)	38 (53.5)
Kp (a+b+)	6 (11.5)	6 (8)	6 (8)	18 (11.2)	11 (26.8)
Kp (a−b+)	27 (51.9)	47 (62.7)	47 (62.7)	99 (61.5)	27 (65.9)
Kp (a+b−)	3 (5.8)	3 (4)	3 (4)	6 (3.7)	2 (4.9)
Kp (a−b−)	16 (30.8)	19 (25.3)	19 (25.3)	38 (23.6)	1 (2.4)
K	2 (3.8)	1 (1.3)	0 (0)	3 (1.9)	0 (0)
k	45 (86.5)	70 (93.3)	34 (100)	149 (92.5)	41 (100)
K-k+	43 (82.6)	69 (92)	34 (100)	146 (90.7)	41 (100)
K−k−	7 (13.4)	5 (6.7)	0 (0)	12 (7.5)	0 (0)
K+k+	2 (3.8)	1 (1.3)	0 (0)	3 (1.9)	0 (0)

HM, hematologic malignancy; CLL, chronic lymphocytic leukemia; CML, chronic myelocytic leukemia; MM, multiple myeloma.
